# The Prognostic Role of Serum β-Trace Protein Levels among Patients on Maintenance Hemodialysis

**DOI:** 10.3390/diagnostics14100974

**Published:** 2024-05-07

**Authors:** Po-Yu Huang, Bang-Gee Hsu, Chih-Hsien Wang, Jen-Pi Tsai

**Affiliations:** 1 Division of Nephrology, Department of Internal Medicine, Dalin Tzu Chi Hospital, Buddhist Tzu Chi Medical Foundation, Chiayi 62247, Taiwan; poyuhs13628@gmail.com; 2 Institute of Medical Sciences, Tzu Chi University, Hualien 97004, Taiwan; 3 Division of Nephrology, Hualien Tzu Chi Hospital, Buddhist Tzu Chi Medical Foundation, Hualien 97004, Taiwan; gee.lily@msa.hinet.net (B.-G.H.); wangch33@gmail.com (C.-H.W.); 4 School of Medicine, Tzu Chi University, Hualien 97004, Taiwan

**Keywords:** β-trace protein, diabetes mellitus (DM), hemodialysis, mortality

## Abstract

Cardiovascular (CV) diseases are the most commonly encountered etiology of mortality in patients having kidney failure. β-Trace protein (BTP) is a biomarker of glomerular filtration function as well as a potential predictor of adverse CV outcomes. This study aimed to determine the prognostic value of BTP in patients on chronic hemodialysis (HD). A total of 96 patients undergoing HD were enrolled. Baseline variables were collected, and the patients were tracked for 3 years. Twenty-five patients died at 3 years. Those who experienced mortality were noted to have higher serum concentrations of BTP and a higher incidence of diabetes mellitus (DM). The area under the receiver operating characteristic curve for serum BTP distinguishing mortality from survival was 0.659 (95% confidence interval [CI], 0.555–0.752; *p* = 0.027). After the adjustment of variables potentially affecting survival rates, BTP levels above the median (adjusted hazard ratio [aHR]: 2.913, 95% CI, 1.256–6.754; *p* = 0.013), the presence of DM (aHR: 2.474, 95% CI, 1.041–5.875; *p* = 0.040), and low serum albumin (aHR: 0.298, 95% CI, 0.110–0.806; *p* = 0.017) independently correlated with survival in HD patients. Serum BTP is a novel biomarker for predicting overall outcomes in HD patients.

## 1. Introduction

Cardiovascular (CV) diseases remain the most common cause of fatality in patients with impaired renal function, including those receiving lifelong dialysis treatment, and mortality attributed to CV diseases is much higher in these populations than in those without kidney dysfunction [1,2,3]. According to recent epidemiological data on the Taiwanese population, the 5-year cumulative survival proportion of chronically dialyzed patients was approximately 55.6% [4]. Besides diabetes and high blood pressure, patients having chronic kidney disease (CKD) also possess non-traditional, kidney-specific CV risk factors, including chronic inflammation, albuminuria, disturbed calcium and phosphorus homeostasis, uremic toxins, oxidative stress, and vascular calcifications [2,5]. Many studies have identified several factors that potentially predict adverse CV outcomes and overall survival of patients on maintenance hemodialysis (HD), including vascular status (aortic stiffness, ankle–brachial index, and extent of abdominal aorta calcification) [6,7,8], nutrition markers (albumin, creatinine, and lipid profiles) [9], and serum levels of galectin-3 [10], sclerostin [11], and uremic toxins (p-cresol sulfate, indoxyl sulfate, and carboxymethyllysine) [12,13,14].

Since many of the uremic molecules were highly protein-bound and have large molecular weights, the serum concentrations were much higher in patients with end-stage kidney disease (ESKD), particularly when patients lose the residual kidney functions [15,16]. The retention of the uremic waste products gives rise to a negative impact on the CV system through the mediation of endothelial malfunction, induction of inflammatory cell hyperfunction, and increased thrombogenicity [17].

β-trace protein (BTP), a glycoprotein belonging to the lipocalin superfamily, catalyzes prostaglandin D2 from prostaglandin H2 and is an endogenous glomerular filtration marker [18,19]. BTP can assess residual kidney function in acute and chronic kidney failure and ESKD; however, BTP is poorly hemodialyzed [20,21,22,23]. Moreover, BTP has implications in the pathobiology of CV morbidities. An animal study on mice found that a hypoxic environment upregulated BTP expression in the myocardium [24]. BTP protects against atherogenesis, inflammation, blood vessel thrombosis, and vascular endothelial cell apoptosis [23,25]. Regarding the clinical aspect, the results of the correlation between serum BTP levels and adverse sequelae are conflicting, depending on the populations recruited. Among patients being hospitalized due to acute decompensated heart failure, higher blood BTP levels were significantly associated with poorer overall survival at one year and a higher likelihood of repeat admission for heart failure [26]. A cohort study recruiting patients with atrial fibrillation and on chronic anticoagulation concluded that higher plasma BTP levels were correlated with increased risk for embolic events and overall death, independent of well-established risk variables included in the CHAD_2_DS_2_-VASc scoring system [27]. In contrast, in patients presenting acutely to the emergency healthcare system with a diagnosis of acute coronary syndrome, a serial checkup of serum BTP levels did not predict the poor prognosis [28].

Up to the present, the association between serum BTP and overall survival in HD patients has not been examined extensively. This investigation aimed to evaluate the association of baseline serum BTP concentrations with overall survival rate at three years in patients on maintenance HD treatment.

## 2. Materials and Methods

### 2.1. Study Participants

**Figure 1:**
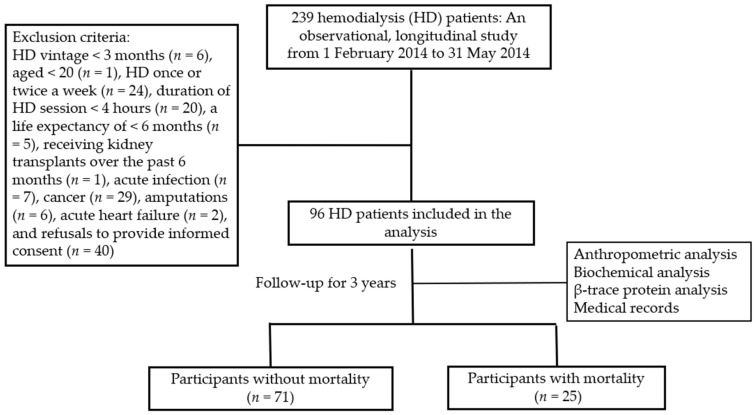
The flowchart of the cohort study on patients undergoing chronic hemodialysis therapy.

This observational, longitudinal research performed in a medical center in Hualian, Taiwan from February 2014 to May 2014 recruited patients receiving chronic HD for over 3 months. Figure 1 represents the flowchart of the cohort study. Patients aged 20 and older received hemodialysis thrice a week for 4 h using a disposable polysulfone dialysis membrane (FX high-flux dialyzer; Fresenius Medical Care, Bad Homburg, Germany). Individuals with a life expectancy of less than 6 months, a renal transplantation surgery within the past 6 months, ongoing infectious diseases, cancer, amputations, acute heart failure, and refusals to complete the informed consent process were excluded from this study. Ninety-six patients on maintenance HD were enrolled. This study was thoroughly reviewed and approved by the Research Ethics Committees of Hualien Tzu Chi Hospital, Buddhist Tzu Chi Medical Foundation (IRB103-136-B).

The same experienced staff used regularly calibrated mercury sphygmomanometers and correctly sized cuffs to measure the systolic blood pressures (SBPs) and diastolic blood pressures (DBPs) of each participant consecutively for three times, and the readings were averaged and then documented. A diagnosis of hypertension was made when the SBP was ≥140 mm Hg, the DBP was ≥90 mm Hg, and/or the medical records revealed the usage of antihypertensive agents (including angiotensin receptor blockers, calcium channel blockers, and beta-adrenergic blockers) over the past two weeks. Patients fulfilled the diagnostic criteria for diabetes mellitus (DM) if they were taking chronic antidiabetic regimens and/or if the fasting glucose level equaled or exceeded 126 mg/dL. The data on the prescription of statins, fibrates, and aspirin for HD patients were also retrieved from the medical charts.

Furthermore, we used the baseline clinical and biochemical variables to estimate the patients’ 10-year risk of atherosclerotic cardiovascular disease (ASCVD) with the ASCVD Risk Estimator Plus provided by the American College of Cardiology [29].

### 2.2. Anthropometric Analysis

With patients standing erect and wearing light clothes, we rounded their heights and weights measurement results to the closest half-centimeter and half-kilogram, respectively. The weights and the subsequently calculated body mass indices (BMIs, equal to weight divided by height squared, in kg/m^2^) were evaluated both directly before and after the HD session.

### 2.3. Biochemical Testing

Patients were asked for an overnight fast before the blood sampling. Immediately prior to the initiation of HD therapy in the midweek, roughly 5 milliliters of blood specimen were drawn from the vascular access. A small fraction of the blood specimen was taken for the analysis of hemoglobin levels via the Sysmex^®^ SP-1000i device (Sysmex American, Mundelein, IL, USA). The remaining samples underwent centrifugation at 3000× *g* for 10 min, and the sera were sent for high-density lipoprotein cholesterol (HDL-C), low-density lipoprotein cholesterol (LDL-C), total cholesterol (TCH), triglyceride (TG), glucose, blood urea nitrogen, creatinine, and albumin measurement with an autoanalyzer (Siemens Healthineers Headquarters, Siemens Healthcare GmbH, Henkestr, Erlangen, Germany). The urea reduction ratio and fractional clearance index for urea (Kt/V), both of which were indicators of small solute clearance, were calculated using the pre- and post-HD blood urea nitrogen levels and the concept of single-pool urea kinetic modeling, respectively. The intact parathyroid hormone level (iPTH) (IBL International GmbH, Hamburg, Germany) and BTP (BioVendor, Karasek, Brno, Czech Republic) were analyzed with validated enzyme-linked immunosorbent assays.

### 2.4. Study Follow-Up

Medical data were used to monitor the patients’conditions for 36 months leading up to 30 June 2017, or until their death. The present study endpoints were defined as all-cause mortality, which comprised hemorrhagic stroke (*n* = 6), acute cerebral ischemia (*n* = 10), acute myocardial infarction (*n* = 5), and severe sepsis with septic shock (*n* = 4). The time interval between the enrollment evaluation and the termination of the study follow-up or establishing the above-specified endpoint was classified as event-free survival.

### 2.5. Statistical Analysis

The continuous variables went through the Kolmogorov–Smirnov test to determine the normality. For those following the normal distribution and then expressed as means ± standard deviations, further analysis and comparison were conducted by the two-tailed independent Student’s *t*-test. Nonnormally distributed continuous variables (HD vintage, TG, glucose, iPTH, BTP, and ASCVD risk percentage) were given as medians with interquartile ranges, and these values were put into the Mann–Whitney U-test to examine the significant differences. The qualitative variables expressed as numbers (percentages) went through the Chi-square test between the groups. The Kaplan–Meier curve illustrates the overall survival as a function of time in patients on maintenance HD over a 3-year follow-up. The receiver operating characteristic (ROC) analytic method was applied to plot the area under the curve as well as to identify the ideal threshold value of BTP to predict death from any cause in hemodialyzed patients. We adopted the univariate and multivariate Cox regression analyses to define the at-risk independent variables for overall fatality. The correlation between log-BTP and the other continuous variables was explored using the Spearman’s correlation analysis; variables showing skewness were logarithmic-transformed in advance. Data were processed with the aid of SPSS software for Windows (version 19.0; IBM Corp., Armonk, NY, USA). *p* values < 0.05 were considered to be statistically significant.

## 3. Results

Table 1 illustrates the baseline clinical variables of patients on maintenance HD. After 3 years of follow-up, 25 patients (26%) died and 71 (74%) of the total were alive. Patients with mortality, when compared to those without mortality at 3 years, were significantly more advanced in age (*p* = 0.024), had a significantly higher proportion of DM (*p* = 0.006), had significantly higher serum BTP (*p* = 0.019) and glucose (*p* = 0.032) concentrations, had significantly lower serum albumin (*p* = 0.002) and creatinine (*p* = 0.001), and had significantly higher ASCVD risk scores (*p* = 0.033). The differences did not reach the statistical significance regarding the gender, percentage of hypertension diagnosis, duration of HD therapy, BMI before and after HD, SBP, DBP, hemoglobin levels, lipid profiles, blood urea nitrogen, calcium, phosphorus, and iPTH between the two groups. Solute clearance indices, including Kt/V and urea reduction rate, as well as the percentage of different antihypertensives, lipid-lowering drugs, or antiplatelet therapy, did not differ between the groups.

**Figure 2:**
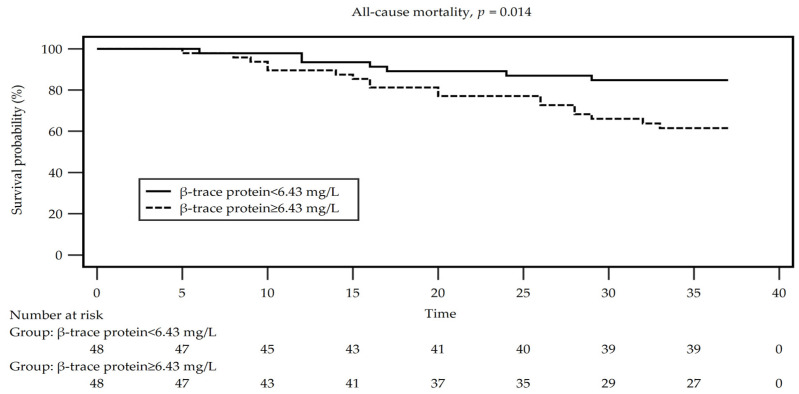
Kaplan–Meier analysis regarding the association β-trace protein (higher versus lower than the median value) with overall survival among patients on maintenance hemodialysis.

Figure 2 displays the Kaplan–Meier survival analysis comparing overall survival between HD patients with serum BTP levels above the median and those with BTP levels below the median. Patients with BTP concentrations lower than the median had significantly greater survival at 36 months than participants with higher BTP levels (*p* = 0.014 for the log-rank test).

**Figure 3:**
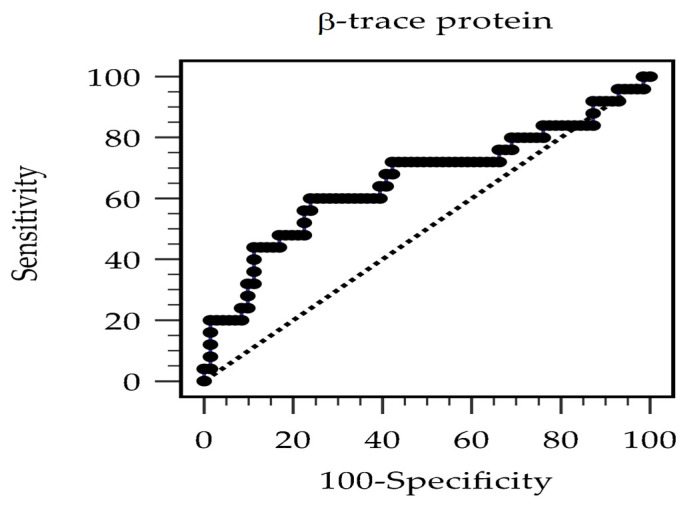
The area under the receiver operating characteristic curve discloses the capability of β-trace protein level in the prognostication of hemodialysis patients.

The area under the ROC curve for serum BTP in distinguishing the dead from the alive at 36 months was 0.659 (95% confidence interval [CI], 0.555–0.752; *p* = 0.027) (Figure 3). The optimal cutoff was 7.102 mg/L, with a sensitivity and specificity of 60.0% and 76.1%, respectively.

Cox proportional hazards models (Table 2) concluded that advancing age (*p* = 0.038), DM (*p* = 0.007), BTP levels above the median (*p* = 0.005), lower albumin concentrations (*p* = 0.001), higher plasma glucose levels (*p* = 0.035), lower serum creatinine levels (*p* = 0.001), and higher ASCVD risk scoring (*p* = 0.001) were potential risk factors for death from any etiology in HD patients. After adjusting variables significantly correlated with survival with the exception of ASCVD risk score, multivariate regression analysis identified DM, higher BTP levels, and hypoalbuminemia as independent prognostic indicators. The adjusted hazard ratio (aHR) for BTP levels above the median was 2.913 (95% CI, 1.256–6.754; *p* = 0.013), the aHR for DM was 2.474 (95% CI, 1.041–5.875; *p* = 0.040), and the aHR for albumin was 0.298 (95% CI, 0.110–0.806; *p* = 0.017).

Table 3 represents the Spearman correlation analytic process between serum log-BTP and clinical and biochemical variables. There was a significant, positive correlation between serum log-BTP level and log-TG (*r* = 0.215, *p* = 0.036), and a significant, inverse correlation between log-BTP and albumin (*r* = −0.235, *p* = 0.021).

## 4. Discussion

The primary finding of this study is that serum BTP levels, underlying DM, and hypoalbuminemia are independent prognostic predictors in patients receiving maintenance HD. Other variables including age, creatinine, and glucose blood levels also correlate with survival in these patients but the significance was attenuated after adjustment.

The molecular weight of BTP was around 23–29 kDa, depending on the status of N-glycosylation as a part of posttranslational protein modification. Circulating BTP is postulated to originate from the central nervous system [18]. Because BTP is a filtration marker, one of the important clinical uses of serum BTP is estimating the glomerular filtration rate (GFR). In addition to commonly used markers recommended by clinical practice guidelines, namely cystatin C and creatinine, several BTP-based estimated GFR formulas have been proposed in a study enrolling patients with the receipt of a kidney transplant [30]. Similar to other novel biomarkers, such as neutrophil gelatinase-associated lipocalin, BTP can be detected and measured in the blood and urine, and is also an indicator of acute and chronic renal dysfunction and renal tubular injury [31,32].

Besides acting as a measure of kidney function, BTP also has physiological and pathophysiological effects on the CV system. BTP is also called lipocalin-type prostaglandin D synthase (L-PGDS), which catalyzes the conversion of prostaglandin H2 to prostaglandin D2. Prostaglandin D2 contributes to vasodilation, inhibits platelet aggregation, and mediates inflammation [25,33]. Within the carotid atherosclerotic plaques, the differential expression of L-PGDS and type 1 membrane-bound prostaglandin E synthase was associated with plaque instability and a propensity for cerebrovascular accidents [34]. Compared with wild-type mice, mice lacking L-PGDS had more severe atherosclerotic plaque lesions and higher inflammatory cytokine expression, including monocyte chemoattractant protein-1 and interleukin-1β [35]. Prostaglandin D2 is further transformed into 15-deoxy-delta 12,14-prostaglandin J2, which acts as a potent activator of peroxisome proliferator-activated receptor γ, downregulates molecular signaling linked to nuclear factor kappa B (NF-κB), and impedes the activity of nitric oxide synthase [36]. The single nucleotide polymorphisms in the L-PGDS gene were noted to influence the extent of atherosclerotic lesions [37]. To summarize, BTP and its metabolites can affect atherosclerotic lesion stability and may further modify the clinical course of CV diseases.

Over the past decade, a number of studies have proven the association of the novel biomarker BTP with kidney failure, CV morbidity and mortality, and overall survival among individuals with chronic renal insufficiency [38]. In patients with CKD, BTP independently correlated with increased odds of kidney failure and mortality [39]. A study discovered that BTP levels above 12.7 mg/L prior to kidney transplantation could predict the post-transplant risk of adverse CV complications, including acute myocardial infarction, cerebrovascular accidents, coronary artery disease prompting bypass surgery or intervention, or CV death in kidney transplant recipients [40]. Serum BTP levels, in addition to cystatin C concentrations, positively correlated with all-cause mortality risk in 226 patients diagnosed with non-ST-segment elevation acute coronary syndrome with a median follow-up period of 859 days. The study participants had relatively preserved kidney function [41].

The relationship of BTP and prognostic state in patients with kidney failure has been previously studied [42]. Our study results were consistent with those of an investigation of incident HD patients that concluded that the highest tertile of BTP levels was associated with the greatest risk for all-cause mortality from 503 individuals who were included in a longitudinal cohort trial of incident dialysis patients between 1995 and 1998 and followed up until 2004. Most patients in the study received dialysis therapy for less than three and a half months upon enrollment. In our study, the median duration of dialysis treatment was more than 4 years. Another study explored the role of BTP in 907 patients with type 2 DM receiving HD from the German Diabetes and Dialysis (4D) study, and in 2962 people having undergone coronary angiography from the Ludwigshafen Risk and Cardiovascular Health research. It revealed that patients with the highest quartile of BTP had the greatest risk of overall mortality even after adjusting for confounders, including age, gender, and comorbidities. In addition, a higher serum BTP to creatinine ratio was a better predictor of CV death than BTP alone [43].

Spearman’s correlation analysis was performed to discover the clinical correlations with BTP. Triglyceride was found to be positively correlated with BTP in the present investigation. Previous research has indicated that BTP might have an impact on lipid metabolism. A study on the U.S. population examined the link between BTP and metabolic syndrome. Patients with the highest quartile of serum BTP concentrations have the lowest probability of having hypertriglyceridemia, which was defined as a serum triglyceride level of 150 mg/dL or higher [44]. In addition, L-PGDS knockout mice exhibited a change in glucose utilization and increased expression of genes responsible for lipogenesis [45]. In summary, the mechanisms of BTP in the alteration of lipid homeostasis remain poorly understood, and whether BTP mediates the formation of atherosclerotic plaques and affects survival through the regulation of lipid metabolism is currently not known. Future research is required for further exploration.

This study also showed that DM independently predicted poor outcomes in HD patients. DM is the most common cause of kidney failure. In contrast to nondiabetic individuals, patients with DM have poor clinical prognoses [46,47]. Among diabetic patients on maintenance HD, those with ESKD due to diabetic nephropathy had an even higher risk of death than those with kidney failure not caused by DM [47]. Furthermore, in HD patients, higher plasma glucose concentrations and higher hemoglobin A1c levels also correlated with decreased survival rates [48,49].

Hypoalbuminemia, a critical indicator of malnutrition as well as acute and chronic inflammation, is a well-known risk factor for unfavorable outcomes in patients on HD [50,51]. In addition to the urine albumin to creatinine ratio, serum creatinine had positive associations with all-cause mortality, and serum albumin and estimated GFR had negative associations with all-cause fatality after the adjustment for traditional CV risk variates, as well as for coronary artery calcified plaque in 1220 participants of European Americans with type 2 DM after accounting for subclinical CV diseases [52]. Based on the multivariate analysis in our research, lower albumin level was an independent risk parameter for death, a finding consistent with these previous studies. On the other hand, creatinine, a by-product of skeletal muscle metabolism which reflects muscle mass, did not independently predict mortality in our study patients. Advancing age was associated with higher chance of death but it was confounded by a variety of CV risk factors; as a result, multivariate analysis did not identify age as an independent risk variable.

We employed the ASCVD Risk Estimator Plus to estimate the possibility of the ASCVD occurrence in the subsequent 10 years among patients receiving chronic HD. So far there is limited evidence regarding the clinical utility of novel ASCVD Risk Estimator Plus in ESKD populations; however, in the present investigation, the risk score was found to be positively correlated with a 3-year mortality rate in patients undergoing HD. Framingham Risk Score, SCORE2 risk model, and Pooled Cohort Equations had been used in the assessment of future adverse CV consequences and survival rates in patients with non-dialysis CKD [53,54,55]. Further clinical trials are mandated for the validation of these risk scoring systems in these patients.

There are a number of limitations in this investigation. First, the single-center clinical study recruited a relatively small number of participants. Second, the area under the ROC curve for BTP levels was below 0.7, and the discriminatory power might not be sufficiently high. Third, we did not incorporate the data on urine output because BTP correlated with residual kidney function [42]. Nevertheless, since most of our study participants had undergone dialysis therapy for prolonged periods (median HD vintage being 55.14 months), the residual kidney function is considered negligible. Fourth, we measured the BTP levels in serum only once at baseline; thus, we failed to recognize whether BTP concentrations changed with time. Fifth, we did not assess the inflammatory status of these participants, such as a checkup of C-reactive protein or interleukin levels. Sixth, we did not perform further analysis on the association of BTP with different causes of mortality because of the small patient numbers. Larger longitudinal studies are necessary to bolster our study results.

## 5. Conclusions

Higher serum levels of BTP, along with diabetes and hypoalbuminemia, are independent risk factors for all-cause deaths in patients on HD. Future perspectives may focus on using several uremic molecules and kidney failure biomarkers simultaneously to optimize mortality prediction in these patients.

## Figures and Tables

**Table 1:** Clinical characteristics of the hemodialysis patients with or without mortality.

Variables	All Participants (*n* = 96)	Participants without Mortality (*n* = 71)	Participants with Mortality (*n* = 25)	*p* Value
Age (years)	62.54 ± 12.76	60.80 ± 12.80	67.48 ± 11.49	0.024 *
Female, *n* (%)	48 (50.0)	35 (49.3)	13 (52.0)	0.817
Diabetes mellitus, *n* (%)	39 (40.6)	23 (32.4)	16 (64.0)	0.006 *
Hypertension, *n* (%)	52 (54.2)	37 (52.1)	15 (60.0)	0.496
HD vintage (months)	55.14 (23.28–122.16)	53.4 (21.63–128.1)	59.4 (29.85–104.61)	0.835
Pre-HD BMI (kg/m^2^)	24.91 ± 4.79	25.25 ± 4.74	23.92 ± 4.90	0.234
Post-HD BMI (kg/m^2^)	23.92 ± 4.57	24.29 ± 4.48	22.86 ± 4.75	0.180
Systolic blood pressure (mm Hg)	144.39 ± 26.83	143.03 ± 25.88	148.28 ± 29.57	0.403
Diastolic blood pressure (mm Hg)	77.10 ± 15.52	77.86 ± 15.89	74.96 ± 14.50	0.425
β-trace protein (mg/L)	6.43 (5.88–7.78)	6.36 (5.86–7.08)	7.51 (6.02–8.97)	0.019 *
Hemoglobin (g/dL)	10.54 ± 1.16	10.60 (9.73–11.18)	10.70 (10.30–11.05)	0.485
Total cholesterol (mg/dL)	146.76 ± 34.88	149.80 ± 34.90	138.12 ± 34.04	0.151
Triglyceride (mg/dL)	113.00 (83.25–175.00)	115 (83.25–184)	105 (81.25–129.25)	0.206
HDL-C (mg/dL)	45.00 (38.00–54.75)	47.00 (38.00–56.00)	42.00 (35.50–52.00)	0.279
LDL-C (mg/dL)	111.24 ± 26.57	113.31 ± 26.48	105.36 ± 26.46	0.200
Glucose (mg/dL)	132.50 (106.25–168.75)	128 (103–160.5)	144 (124.5–185.25)	0.032 *
Blood urea nitrogen (mg/dL)	60.64 ± 13.83	61.17 ± 14.10	59.12 ± 13.19	0.527
Creatinine (mg/dL)	9.45 ± 1.95	9.82 ± 1.84	8.37 ± 1.89	0.001 *
Total calcium (mg/dL)	9.02 ± 0.81	9.04 ± 0.82	8.97 ± 0.81	0.723
Phosphorus (mg/dL)	4.66 ± 1.35	4.72 ± 1.37	4.49 ± 1.32	0.480
iPTH (pg/mL)	191.45 (69.33–479.48)	205.2 (77.28–49.43)	160.3 (57.3–522.13)	0.997
Urea reduction rate	0.74 ± 0.04	0.73 (0.70–0.76)	0.76 (0.71–0.78)	0.184
Kt/V (Gotch)	1.35 ± 0.17	1.30 (1.21–1.43)	1.42 (1.25–1.52)	0.179
10-year ASCVD risk (%)	14.00 (4.70–27.33)	10.40 (4.10–22.70)	20.70 (11.65–38.40)	0.033 *
ARB, *n* (%)	29 (30.2)	23 (32.4)	6 (24.0)	0.432
β-blocker, *n* (%)	31 (32.3)	26 (36.6)	5 (20.0)	0.126
CCB, *n* (%)	38 (39.6)	32 (45.1)	6 (24.0)	0.064
Statin, *n* (%)	16 (16.7)	14 (19.7)	2 (8.0)	0.176
Fibrate, *n* (%)	12 (12.5)	9 (12.7)	3 (12.0)	0.930
Aspirin, *n* (%)	67 (69.8)	49 (69.0)	18 (72.0)	0.780

Continuous variables were compared with the Student’s *t*-test if normally distributed (expressed as mean ± standard deviation) and compared with the Mann–Whitney U test if not normally distributed [expressed median (interquartile range)]. Qualitative variables were compared between the two groups using the Chi-square test. Abbreviations: HD, hemodialysis; BMI, body mass index; HDL-C, high-density lipoprotein cholesterol; LDL-C, low-density lipoprotein cholesterol; iPTH, intact parathyroid hormone; Kt/V, fractional clearance index for urea; ASCVD, atherosclerotic cardiovascular disease; ARB, angiotensin-receptor blocker; CCB, calcium-channel blocker. * *p* < 0.05 was considered to be statistically significant.

**Table 2:** Multivariate Cox proportional-hazards regression analysis showing the independent correlates of death in 96 patients on chronic hemodialysis therapy.

Variables	HR	95% CI	*p* Value	aHR	95% CI	*p* Value
Age (years)	1.034	1.002–1.068	0.038 *	1.021	0.985–1.058	0.268
Female/Male	0.948	0.640–1.404	0.790	–	–	–
Diabetes mellitus	3.092	1.365–7.000	0.007 *	2.474	1.041–5.875	0.040 *
Hypertension	1.433	0.644–3.190	0.378	–	–	–
HD vintage (months)	0.998	0.992–1.004	0.592	–	–	–
Pre-HD BMI (kg/m^2^)	0.955	0.875–1.043	0.306	–	–	–
Post-HD BMI (kg/m^2^)	0.945	0.861–1.037	0.230	–	–	–
Systolic blood pressure (mm Hg)	1.007	0.993–1.021	0.346	–	–	–
Diastolic blood pressure (mm Hg)	0.992	0.966–1.018	0.533	–	–	–
β-trace protein						
<6.43 mg/L	1					
≥6.43 mg/L	3.188	1.428–7.115	0.005 *	2.913	1.256–6.754	0.013 *
Hemoglobin (g/dL)	1.195	0.858–1.664	0.292	–	–	–
Total cholesterol (mg/dL)	0.991	0.979–1.003	0.146	–	–	–
Triglyceride (mg/dL)	0.996	0.990–1.002	0.157	–	–	–
HDL-C (mg/dL)	0.983	0.952–1.014	0.280	–	–	–
LDL-C (mg/dL)	0.989	0.972–1.007	0.217	–	–	–
Albumin (g/dL)	0.217	0.087–0.544	0.001 *	0.298	0.110–0.806	0.017 *
Glucose (mg/dL)	1.005	1.000–1.009	0.035 *	1.002	0.997–1.007	0.498
Blood urea nitrogen (mg/dL)	0.992	0.965–1.020	0.590			
Creatinine (mg/dL)	0.693	0.558–0.861	0.001 *	0.787	0.618–1.002	0.052
Total calcium (mg/dL)	0.910	0.558–1.148	0.706	–	–	–
Phosphorus (mg/dL)	0.902	0.673–1.208	0.487	–	–	–
iPTH (pg/mL)	1.001	1.000–1.002	0.222	–	–	–
Urea reduction rate (×100)	1.051	0.960–1.152	0.282	–	–	–
Kt/V (Gotch)	3.373	0.375–30.38	0.278	–	–	–
ASCVD risk	1.033	1.014–1.052	0.001 *	–	–	–
ARB	0.693	0.277–1.737	0.434	–	–	–
β-blocker	0.519	0.195–1.384	0.190	–	–	–
CCB	0.465	0.186–1.164	0.102	–	–	–
Statin	0.430	0.101–1.824	0.252	–	–	–
Fibrate	0.942	0.282–3.145	0.922	–	–	–
Aspirin	1.129	0.471–2.703	0.786	–	–	–

Multivariate Cox proportional-hazards regression analysis is adjusted for age, diabetes mellitus, albumin, fasting glucose, creatinine, and β-trace protein; ASCVD was not considered as the adjusted variable due to concern over interactions. Abbreviations: HR, hazard ratio; CI, confidence interval; aHR, adjusted hazard ratio; HD, hemodialysis; BMI, body mass index; HDL-C, high-density lipoprotein cholesterol; LDL-C, low-density lipoprotein cholesterol; iPTH, intact parathyroid hormone; Kt/V, fractional clearance index for urea; ASCVD, atherosclerotic cardiovascular disease; ARB, angiotensin-receptor blocker; CCB, calcium-channel blocker. * *p* < 0.05 was considered to be statistically significant in the Cox regression analysis.

**Table 3:** Spearman correlation coefficients between serum logarithmic-transformed β-trace protein levels and clinical parameters of 96 patients on hemodialysis.

Variables	Spearman Coefficient of Correlation	*p* Value
Age (years)	−0.174	0.090
Log-HD vintage (months)	−0.125	0.226
Pre-HD BMI (kg/m^2^)	0.124	0.230
Post-HD BMI (kg/m^2^)	0.112	0.279
Systolic blood pressure (mm Hg)	−0.132	0.199
Diastolic blood pressure (mm Hg)	−0.155	0.131
Hemoglobin (g/dL)	0.044	0.674
Total cholesterol (mg/dL)	−0.037	0.718
Log-Triglyceride (mg/dL)	0.215	0.036 *
Log-HDL-C (mg/dL)	−0.105	0.309
LDL-C (mg/dL)	0.037	0.724
Albumin (g/dL)	−0.235	0.021 *
Log-Glucose (mg/dL)	0.045	0.661
Blood urea nitrogen (mg/dL)	−0.041	0.689
Creatinine (mg/dL)	−0.092	0.374
Total calcium (mg/dL)	−0.083	0.424
Phosphorus (mg/dL)	−0.040	0.696
Log-iPTH (pg/mL)	−0.003	0.977
Urea reduction rate	0.024	0.818
Kt/V (Gotch)	0.026	0.802
Log-ASCVD risk	−0.107	0.301

The variables including β-trace protein, HD vintage, triglyceride, HDL-C, glucose, iPTH, and ASCVD risk score showed skewedness and consequently went through the logarithmic transformation prior to the Spearman correlation analysis. Abbreviations: HD, hemodialysis; BMI, body mass index; HDL-C, high-density lipoprotein cholesterol; LDL-C, low-density lipoprotein cholesterol; iPTH, intact parathyroid hormone; Kt/V, fractional clearance index for urea; ASCVD, atherosclerotic cardiovascular disease. * *p* < 0.05 was considered to be statistically significant.

## Data Availability

The data presented in this study are available on request from the corresponding author.
